# Pharmacological intervention of the HMGB1-pCTS-L axis to ameliorate inflammatory diseases

**DOI:** 10.3389/fimmu.2026.1843251

**Published:** 2026-05-08

**Authors:** Weiqiang Chen, Jianhua Li, Xiaoling Qiang, Li Lou, Cassie Shu Zhu, Meihong Deng, Haichao Wang

**Affiliations:** 1 Northwell, New Hyde Park, NY and Feinstein Institutes for Medical Research, Northwell Health, Manhasset, NY, United States; 2 Departments of Emergency Medicine and Molecular Medicine, Donald and Barbara Zucker School of Medicine at Hofstra/Northwell, Hempstead, NY, United States

**Keywords:** HMGB1 (high mobility group box 1), mimetic peptide inhibitor, neutralizing antibodies, procathepsin-L, tetranectin

## Abstract

Dysregulated inflammation, characterized by the uncontrolled release of inflammatory mediators, is central to the pathogenesis of numerous inflammatory diseases, including acute sepsis and chronic rheumatoid arthritis (RA). Despite therapeutic advances in RA, limitations of current anti-inflammatory treatments—such as broad immunosuppression and partial efficacy—highlight an urgent need for novel interventions. This review critically examines the pathogenic roles of High Mobility Group Box 1 (HMGB1) and procathepsin-L (pCTS-L) in dysregulated inflammation, mediated by their interactions with Toll-like receptor 4 (TLR4) and the Receptor for Advanced Glycation End Products (RAGE). Crucially, we highlight the newly established HMGB1-pCTS-L axis, in which HMGB1 directly upregulates pCTS-L expression and release. This axis initiates a delayed yet sustained inflammatory loop, which may predominantly activate the more enduring non-canonical NF-κB pathway. Furthermore, we explore the intricate role of tetranectin (TN), an endogenous HMGB1-binding protein, which inhibits HMGB1 release but paradoxically facilitates HMGB1-induced pyroptosis. Leveraging this complexity, we introduce the TN-derived P2–1 peptide as a highly specific inhibitor of the HMGB1-pCTS-L axis. This peptide binds HMGB1 to prevent its RAGE engagement and subsequent macrophage pyroptosis, without broadly suppressing initial inflammatory cascades. It also specifically inhibits HMGB1-induced pCTS-L expression and release, ameliorating both sepsis and RA even with delayed treatment. Its “disease-triggered” mechanism, selectively targeting extracellular HMGB1 only at pathological sites, promises enhanced safety and precision. This review positions the HMGB1-pCTS-L axis as a critical and therapeutically tractable pathway, with HMGB1- and pCTS-L-inhibiting antibodies and mimetic peptides representing promising next-generation interventions for a spectrum of inflammatory diseases.

## 1 Overview of core inflammatory pathways for therapeutic advancement

Inflammation is a fundamental protective response to infection or injury, characterized in part by the activation of innate immune cells and the production of various proinflammatory mediators. These mediators include tumor necrosis factor (TNF) (1, 2), Interleukins (IL-1) (3), high mobility group box 1 (HMGB1) (4, 5), and Procathepsin-L (pCTS-L) (6, 7). However, when feedback regulatory mechanisms become dysregulated, the uncontrolled production and/or release of these mediators—originally intended for host defense—can instead initiate and perpetuate excessive immune activation and immunosuppression. This contributes significantly to the pathogenesis of various inflammatory diseases, such as sepsis—an acute inflammatory condition responsible for approximately one-fifth of all fatalities annually (8), and rheumatoid arthritis (RA)—a chronic autoimmune disorder impacting 0.5 – 1% of the world’s population (9). Despite failures in clinical sepsis, anti-TNF biologics have become cornerstone therapies for RA, uncovering crucial mechanisms of inflammatory diseases (10–14). This historical success also underscores how models of acute inflammation (e.g., sepsis) can effectively reveal core pathways underpinning chronic autoimmune conditions (e.g., RA) (14). Therefore, a comprehensive understanding of the intricate roles of various inflammatory mediators is crucial for elucidating intricate disease mechanisms and developing novel therapeutic interventions.

## 2 Discovery of HMGB1 as a late-acting DAMP in sepsis

**Figure 1:**
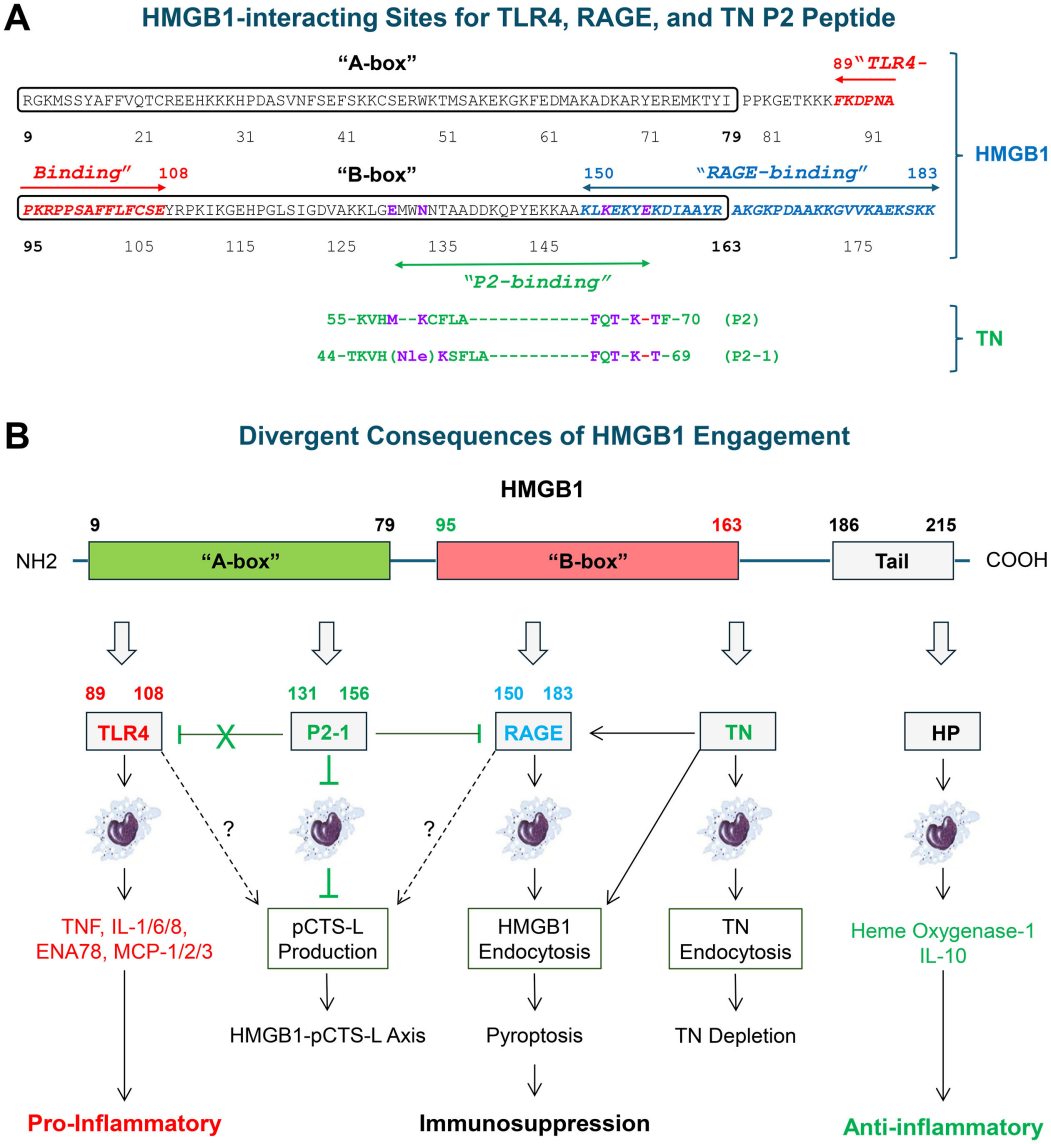
Divergent modulation of HMGB1 functions through distinct molecular interactions. **(A)** Interfacial residues of human HMGB1 for interacting with TLR4, RAGE, or TN P2. The binding regions for TLR4 (residues 89–108) and RAGE (residues 150–183) are highlighted in italicized red and blue text, respectively. Key P2-interacting residues are highlighted in non-italicized purple text. **(B)** Divergent functional outcomes stemming from HMGB1’s interaction with various proteins. HMGB1 engages TLR4 and RAGE receptors, leading to the induction of cytokine/chemokine production and macrophage pyroptosis, respectively. Conversely, some endogenous proteins, such as HP, can bind to HMGB1 to induce anti-inflammatory IL-10. Similarly, TN can sequester HMGB1 to facilitate the endocytosis of HMGB1/TN complexes, and subsequently induce macrophage pyroptosis and immunosuppression. In contrast, a TN-derived P2–1 peptide binds HMGB1 to prevent its interaction with a pro-endocytic receptor, RAGE, thereby suppressing macrophage pyroptosis and HMGB1-mediated pCTS-L upregulation.

As a member of the high mobility group (HMG) protein family (A1, B1/2, N1/2), HMGB1 is the most ubiquitous and evolutionarily conserved nuclear protein, sharing remarkable sequence identity across species (15). Its nuclear role involves chromatin organization and gene expression modulation through interaction with DNA (via Box-A and Box-B, **Figure 1A**) and transcription factors. This intrinsic function underscores its fundamental importance in maintaining both genomic integrity and cellular functions under normal physiological conditions (15). In addition to its intracellular functions, HMGB1’s role extends far beyond the confines of the nucleus. Once released, extracellular HMGB1 acts as a potent damage-associated molecular pattern (DAMP), particularly in inflammatory conditions like sepsis (4, 5, 16, 17) and RA (18–22).

### 2.1 Active secretion and passive release of HMGB1 by immune cells

Our seminal research in 1999 revealed that bacterial endotoxins (e.g., lipopolysaccharide, LPS) and early cytokines (e.g., TNF) stimulate innate immune cells to release HMGB1 with delayed kinetics, thereby positioning it as a late-acting mediator of endotoxemia (4). Extracellular HMGB1 acts as a prototypical DAMP, intensifying inflammation and contributing to the pathology of sepsis (4, 5, 16, 17) and RA (18–22). In response to LPS and pro-inflammatory cytokines (e.g., TNF) (4), HMGB1 can be released via active secretion. However, its secretion bypasses classical endoplasmic reticulum (ER)-Golgi exocytotic pathways, but relies on acetylation (23), lactylation (23), or phosphorylation (24, 25) of HMGB1’s nuclear localization sequence (NLS). These post-translational modifications facilitate HMGB1 export from the nucleus, enabling cytoplasmic trafficking and non-classical (exosome-like) secretion (23, 26–29). Interestingly, a recent study revealed that HMGB1 can interact with other proteins to form an interactome in the cytoplasm (30), whose function in regulating HMGB1 release awaits future investigation.

In addition, HMGB1 can be passively released via necrosis (31) or pyroptosis (32), a lytic form of programmed cell death driven by canonical (NLRP3/ASC/CASP-1) and non-canonical (CASP-11/4/5) inflammasome pathways (33). For instance, bacterial endotoxins and cytokines [e.g., type I/II interferons and serum amyloid A (SAA)] can promote inflammasome activation (34–37), leading to hemichannel (e.g., Pannexin 1 and Connexin 43) expression (38–40), and the formation of gasdermin D pores that causes membrane rupture and extracellular release of HMGB1 (32, 41).

### 2.2 Extracellular HMGB1 drives dysregulated inflammation and immunosuppression

Once outside the cell, HMGB1 exerts its pathogenic effects through interacting with various receptors, dictating the nature and intensity of the inflammatory response. At lower concentrations, HMGB1 binds with high affinity (equilibrium dissociation constant K_D_ = 22.0 nM) to the Toll-like receptor 4 (TLR4) via residues 89–108 (**Figures 1A, B**) (42–44). This interaction boosts the production of cytokines and chemokines by macrophages (45), neutrophils (46), and endothelial cells (47), thereby escalating inflammation. At higher concentrations, HMGB1 also binds RAGE predominantly via residues 150–183 (**Figures 1A, B**) (15), thereby promoting RAGE-dependent endocytosis of HMGB1 and its associated cargos. For instance, when complexed with microbial pathogen-associated molecular patterns (PAMPs, e.g., CpG-DNA, LPS, and SARS-CoV-2 spike protein), HMGB1 can facilitate cellular uptake of these PAMPs (17, 48) via RAGE-dependent endocytosis (49, 50). This cellular trafficking enhances the access of these PAMPs to their intracellular pattern recognition receptors like TLR9 (51–53) or CASP-11 (17), which in turn promotes hyperinflammation or pyroptosis (17, 54). Even in the absence of PAMPs, HMGB1 itself can also trigger pyroptosis of innate immune cells (**Figure 1B**) (54) and subsequently lead to immunosuppression (55) that hinders pathogen clearance (56). Collectively, these actions position extracellular HMGB1 as a late-acting DAMP, capable of shifting the balance towards either excessive inflammation or profound immunosuppression (**Figure 1B**) (4, 5, 57), both of which contribute to the pathogenesis of sepsis and RA. Although HMGB1, particularly when fully reduced (all-thiol state of its three Cys residues) (58), can also bind to TLR2 (43, 44), the role of TLR2 in HMGB1-elicited inflammatory responses remains an intriguing area for further investigation.

While HMGB1 is classically recognized as a pro-inflammatory DAMP, its significant role in the immunosuppressive phase of sepsis, often following an initial hyperinflammatory response, is increasingly recognized. HMGB1 contributes to immune suppression through several mechanisms. Although initial HMGB1-TLR4 signaling is typically pro-inflammatory, chronic and sustained activation of these pathways can lead to immune cell exhaustion, particularly in macrophages and T cells (59, 60). This prolonged exposure can induce epigenetic modifications that suppress the gene expression of pro-inflammatory mediators, pushing cells toward a state of hyporesponsiveness or anergy. Furthermore, HMGB1 often forms complexes with LPS and other PAMPs (e.g., lipoteichoic acid, LTA) (61). Persistent TLR2/TLR4 stimulation, even by these HMGB1-PAMP complexes, is a key mechanism driving endotoxin tolerance in macrophages (62), rendering them refractory to subsequent stimulation and thereby contributing to immunosuppression. HMGB1 has also been shown to promote the recruitment, expansion, and activation of Myeloid-Derived Suppressor Cells (MDSCs) (63), which are potent suppressors of T cell function and significantly contribute to sepsis-induced immunosuppression. While less directly studied than MDSCs, HMGB1’s inflammatory signals might indirectly contribute to the generation or activation of regulatory T cells (Tregs) in certain contexts (64), which are also crucial mediators of immune tolerance and suppression.

As aforementioned, extracellular HMGB1 can also induce apoptosis (65) and pyroptosis (17, 54) of innate immune cells (e.g., macrophages). This loss of innate immune cells severely compromises the host’s ability to mount an effective immune response against secondary infections, a hallmark of sepsis-induced immunosuppression. Although pyroptosis is most prominently studied in macrophages, the internalization of HMGB1, potentially in complex with other molecules (like PAMPs), could likely induce pyroptosis in other types of cells beyond macrophages, including endothelial and various epithelial cells, which could significantly contribute to the widespread organ damage observed in sepsis. Thus, further investigations are needed to precisely delineate the specific cell types, receptors, and downstream molecular pathways involved in HMGB1-mediated pyroptosis and immunosuppression.

Recently, a study revealed that mimetic peptides corresponding to HMGB1 residues 89–98 and 160–169 competitively inhibited its interaction with TLR4 and RAGE, thereby conferring protection against lethal sepsis (**Table 1**) (66). Similarly, an 18-aa peptide (OP18), designed based on a TLR4 extracellular domain peptide and an RGD motif, effectively sequestered multiple DAMPs that included HMGB1 (67). This peptide promoted the phagocytosis of several DAMPs via α_v_β_3_-integrin-mediated endocytosis, consequently protecting against sepsis by reducing systemic DAMPs (**Table 1**) (67).

**Table 1:** Therapeutic potential of HMGB1-, pCTS-L-, and TN-specific neutralizing antibodies and/or mimetic peptides.

Pharmacological agents	Mechanism of action	Therapeutic efficacy	References
HMGB1 A-box antagonist	Competes with HMGB1 for TLR4 and RAGE interaction	Protective against lethal sepsis and RA	(5)
HMGB1 mimetic peptides (residues 89–98 and 160-169)	Compete with HMGB1 for TLR4 and RAGE interaction	Protective against lethal sepsis	(66)
TLR4 mimetic peptide	Binds and sequesters multiple DAMPs (e.g., HMGB1)	Protective against lethal sepsis	(67)
HMGB1-reactive mAbs	Neutralize the pro-inflammatory activities of HMGB1	Protective against lethal sepsis and RA	(5, 16, 19, 22, 77)
pCTS-L P13 peptide (residues 194-214)-reactive mAbs	Block pCTS-L interactions with TLR4 and RAGE receptors	Protective against lethal sepsis and RA	(6, 100)
HMGB1-binding proteins (e.g., HP)	Promote anti-inflammatory cytokine (IL-10) production	Protective against lethal sepsis	(117)
TN P5-reactive mAbs	Disrupt TN/HMGB1 interaction, block HMGB1 endocytosis, macrophage pyroptosis, and potentially mitigate immunosuppression	Protective against lethal sepsis and endotoxemia	(121, 134)
TN P2-reactive mAbs		Worsens the outcome of sepsis	(121)
TN protein	Sequesters circulating HMGB1, thereby removing it from the circulation, and enhances HMGB1 endocytosis and macrophage pyroptosis	Protective against lethal endotoxemia and sepsis at physiological doses	(121)
TN mimetic P2 peptide and derivatives	Disrupt HMGB1-RAGE interaction, and inhibits HMGB1 endocytosis and pCTS-L expression	Protective against lethal sepsis and RA	(100)

### 2.3 HMGB1 as a late mediator of lethal sepsis with a broader therapeutic window

**Figure 2:**
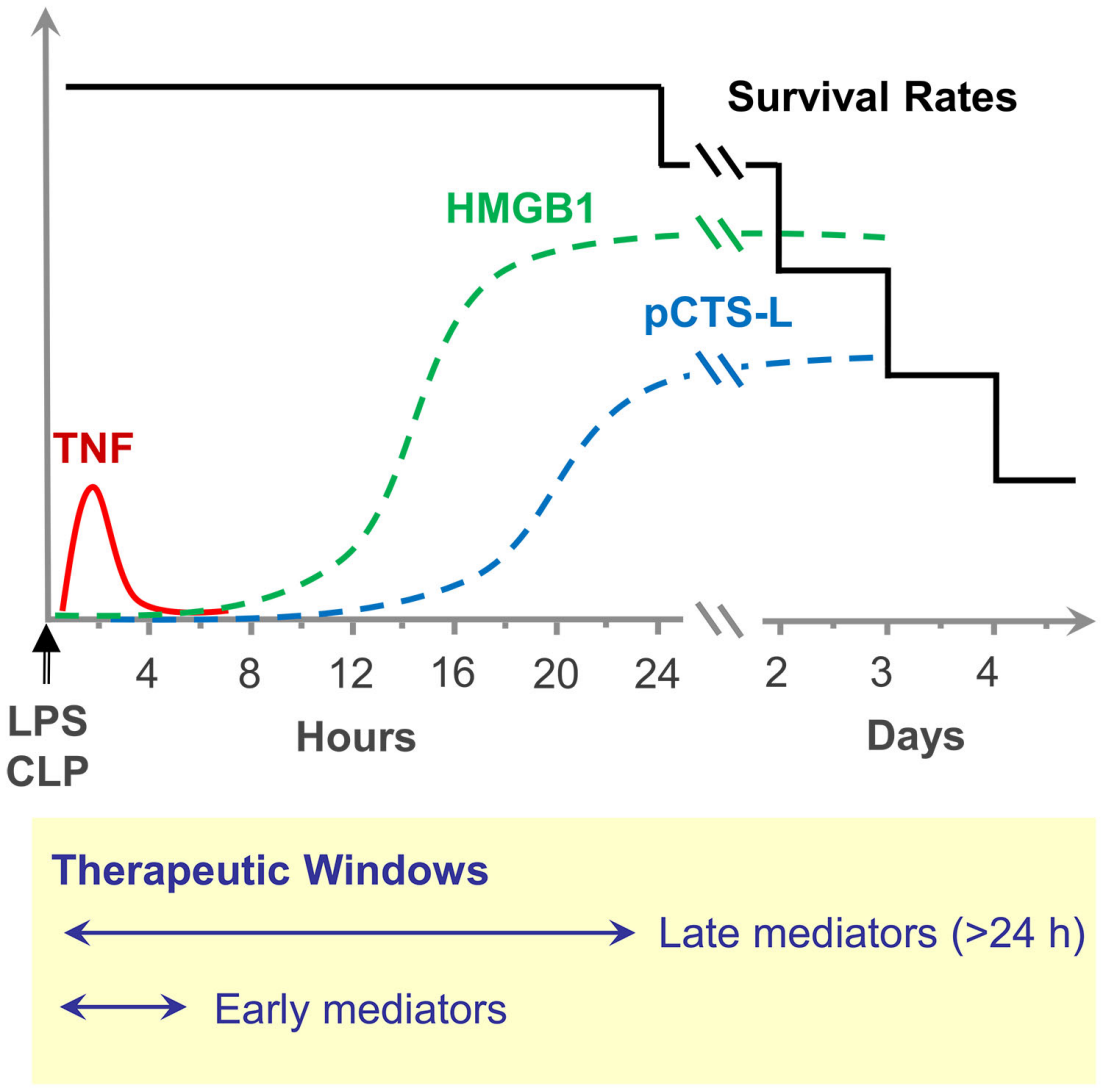
Temporal kinetics of early and late mediators in lethal systemic inflammation. This graph illustrates the distinct temporal release patterns of key inflammatory mediators following an acute systemic inflammatory challenge, such as endotoxemia (induced by intraperitoneal injection of lipopolysaccharide, LPS) or sepsis (induced by Cecal Ligation and Puncture, CLP). Early Mediators such as TNF (red solid line) are rapidly released, peaking within a few hours post-insult before quickly returning to baseline. In contrast, late mediators like HMGB1 (green dashed line) begin to rise later, typically around 8–16 hours, and remains significantly elevated for an extended period, often persisting for several days. Another mediator, pCTS-L (blue dashed line) shows an even later increase, becoming prominent after 24 hours and maintaining high levels for days. The inset box highlights the critical concept of distinct therapeutic windows. Interventions targeting rapidly appearing early mediators like TNF would be most effective within the first few hours. Conversely, therapeutic strategies aimed at sustained late mediators, such as HMGB1 and pCTS-L, would be relevant beyond 24 hours and into the later phases of the disease.

HMGB1 is uniquely positioned as a *late-acting* mediator in sepsis, distinct from rapidly peaking early pro-inflammatory cytokines (e.g., TNF and IL-1). In murine endotoxemia, circulating HMGB1 emerges at 8 hours and peaks between 16–32 hours (**Figure 2**) (4). Similarly, during polymicrobial sepsis, HMGB1 levels rise around 18 hours, reaching a peak between 18–36 hours (**Figure 2**) (5). This delayed appearance of systemic HMGB1 correlates with the onset of animal mortality in experimental endotoxemia and sepsis, thereby distinguishing it from other rapidly peaking pro-inflammatory cytokines (e.g., TNF, **Figure 2**) (68). Consistent with this, HMGB1-neutralizing antibodies or peptide antagonists (e.g., HMGB1 A-box, **Figure 1A**) rescued mice from lethal sepsis (**Table 1**) (5, 16), even when administered up to 24 hours after disease onset. These findings firmly establish HMGB1 as a late mediator, offering a crucial and extended therapeutic window for intervention in severe inflammatory conditions like sepsis (7, 57, 68).

### 2.4 HMGB1 as a pathogenic mediator of rheumatoid arthritis

RA is a chronic autoimmune disease characterized by debilitating inflammation of the synovial joints, leading to progressive cartilage and bone destruction (9). While RA’s etiology is complex and multifactorial (9), the release of HMGB1 occupies a pivotal role in perpetuating chronic inflammation and tissue damage (69, 70). For instance, HMGB1 levels are elevated in the synovial fluid and inflamed tissues of patients or animals with RA (21, 71–73). Its release can occur through passive leakage from necrotic cells due to inflammatory tissue damage, or active secretion by activated immune cells (e.g., macrophages, neutrophils, and dendritic cells) or synovial fibroblasts.

Intraarticular HMGB1 injection induced synovitis characteristic of RA, contributing to the proteolytic degradation of cartilage (20). Its ability to form complexes with other DAMPs, such as DNA or nucleosomes, can also augment its pro-inflammatory potential and contribute to maintaining autoantibody production by B cells (74, 75). Beyond amplifying inflammation, HMGB1 also directly causes pain (76) and contributes to tissue destruction and aberrant cellular processes within the RA joint (75). For instance, HMGB1 can promote the proliferation and survival of synovial fibroblasts, transforming them into an aggressive, tumor-like pannus that invades and destroys articular cartilage and subchondral bone (20). In contrast, HMGB1-neutralizing antibodies or peptide antagonists (e.g., the A-box) reduced disease severity in a collagen-induced arthritis model (**Table 1**) (19, 22, 77), supporting a pathogenic role for extracellular HMGB1 in RA.

## 3 Procathepsin-L as an inducible late-acting mediator in lethal sepsis

Cathepsin L (CTS-L) is a highly conserved cysteine protease (78, 79) crucial for antigen processing (80). Synthesized as an inactive procathepsin L (pCTS-L), it matures into its active form in the lysosomes. Inflammatory stimuli like LPS and IFN-γ induce pCTS-L expression and release from immune cells (81–83). Similarly, we discovered that both LPS and SAA induced the expression and secretion of pCTS-L in primary human peripheral blood mononuclear cells (PBMCs) (6). Unlike HMGB1, pCTS-L contains a signal peptide, enabling its efficient secretion via the classical ER–Golgi pathway.

### 3.1 Induction of *Ctsl* expression in sepsis

In experimental sepsis, *Ctsl* mRNA expression significantly increases in the heart, intestine, kidney, liver, lung, and spleen (6). Circulating pCTS-L protein levels rise with delayed kinetics, peaking around 24–32 hours post-insult in CLP-induced sepsis (**Figure 2**), coinciding with mortality onset. In human sepsis patients, plasma pCTS-L is elevated, correlating with Sequential Organ Failure Assessment (SOFA) scores and inflammatory cytokines. This evidence points to a sustained systemic accumulation of pCTS-L in both experimental and clinical sepsis, suggesting its possible involvement in disease progression (6).

### 3.2 Extracellular pCTS-L signals via TLR4 and RAGE to amplify inflammation

Like HMGB1, recombinant pCTS-L binds with high affinity to TLR4 (equilibrium dissociation constant K_D_ = 20 nM) and RAGE (K_D_ = 3.5 nM), and potently induces the production of cytokines (e.g., IL-6 and TNF) and chemokines (e.g., RANTES, MCP-1, ENA-78/LIX, IL-8, GRO-α/KC, and GRO-α/β/γ) in human PBMCs (6). Genetic deletion of these receptors attenuated pCTS-L-induced cytokine/chemokine release, and conferred resistance to pCTS-L-driven inflammation *in vivo*. Furthermore, pCTS-L upregulates pro-caspase-11 and promotes CASP-11 maturation in a TLR4/RAGE-dependent manner (6). This may create a feed-forward loop activating non-canonical inflammasomes and pyroptosis. *In vivo*, pCTS-L induced hepatic fibrinogen-α/β/γ expression and sinusoidal thrombosis (6), which are partly attenuated by double-knockout of both TLR4 and RAGE. Collectively, TLR4 and RAGE, along with yet-to-be identified receptors, are responsible for transmitting the extracellular signaling of pCTS-L to hyperinflammation and pyroptosis-associated immunosuppression under pathological conditions.

### 3.3 Pathogenic role of pCTS-L in sepsis and RA

To assess the pathogenic role of extracellular pCTS-L in inflammatory diseases, we typically employed both genetic and pharmacological approaches. Genetic ablation of *Ctsl* mitigates CLP-induced liver injury and other pathologies, suggesting pCTS-L’s pathogenic role in sepsis. Although knockout approaches have been widely used to characterize gene functions, they often complicate the interpretation of extracellular mediators in the context of other cell-intrinsic roles. For example, extracellular HMGB1 is pathogenic in sepsis (5, 7, 57), but its genetic loss increases animal’s susceptibility to infections (84) and injuries (85), highlighting its vital intracellular functions even under pathological conditions.

Recognizing the complexities of knockout models for extracellular mediators, we initially generated polyclonal antibodies against murine pCTS-L. These polyclonal antibodies protected mice from lethal CLP sepsis, in part by attenuating extracellular pCTS-L-mediated systemic inflammation (6). Following this, we generated monoclonal antibodies (mAbs) against murine and human pCTS-L to assess their therapeutic efficacy. Three mAbs targeting a unique epitope (residues 194–214) of pCTS-L similarly improved animal survival, even with delayed administration (**Table 1**). These protective mAbs attenuated pCTS-L-induced cytokine/chemokine production in human PBMCs, and reduced sepsis-induced systemic accumulation of surrogate markers (e.g., IL-6, KC/GRO-α, and MIP-2/GRO-β) (6). Similarly, several chemical inhibitors (e.g., progesterone and lanosterol) selectively blocked pCTS-L-induced cytokine/chemokine production, and conferred protection against lethal sepsis (86, 87). Collectively, these findings demonstrated a pathogenic role of extracellular pCTS-L accumulation in lethal sepsis (6, 7).

Sepsis and RA share a common immunopathological pathway, characterized by the uncontrolled production and release of key pro-inflammatory cytokines (e.g., TNF, IL-1, and IL-6) and DAMPs (e.g., HMGB1) (4, 5, 18–22, 88, 89). While numerous clinical interventions for sepsis have unfortunately failed, anti-TNF mAbs have nonetheless emerged as cornerstone therapies for RA patients (10–14). Nonetheless, anti-TNF therapies only offer partial efficacy for some patients (90), and can adversely increase susceptibility to infections (91). Given these limitations, we explored the pathogenic role of pCTS-L and therapeutic efficacy of its neutralizing antibodies in a collagen antibody-induced arthritis (CAIA) model.

The pathogenic role of pCTS-L in RA was supported by its elevation in experimental (92–94) and clinical arthritis (95–97), as well as findings that its genetic deletion or pharmacological suppression attenuated experimental arthritis (98, 99). Consistently, we demonstrated that a pCTS-L-neutralizing mAb2 significantly lessened arthritis severity and pain sensitivity in the CAIA model (**Table 1**) (100). These findings firmly establish pCTS-L as an exceptionally promising therapeutic target for both sepsis (6) and RA (100), potentially offering an alternative therapeutic avenue distinct from existing anti-TNF biologics.

### 3.4 Protective mechanisms of pCTS-L-neutralizing antibodies

Mechanistically, these protective antibodies significantly reduced pCTS-L’s binding affinity for key receptors, TLR4 and RAGE (**Table 1**) (6), as evidenced by an almost 10- and 55-fold increase in the equilibrium dissociation constant (K_D_) for TLR4 and RAGE, respectively. Molecular docking analyses suggest that the P-13 epitope region of pCTS-L-neutralizing mAbs is situated within a hydrophobic pocket on TLR4 and is adjacent to the RAGE V domain in the ligand-receptor complexes (6). This suggests a model wherein these pCTS-L-neutralizing mAbs competitively block its receptor engagement, thereby blunting downstream inflammatory response and protecting mice against sepsis and RA.

## 4 Comparison of HMGB1 and pCTS-L immunomodulatory properties

**Figure 3:**
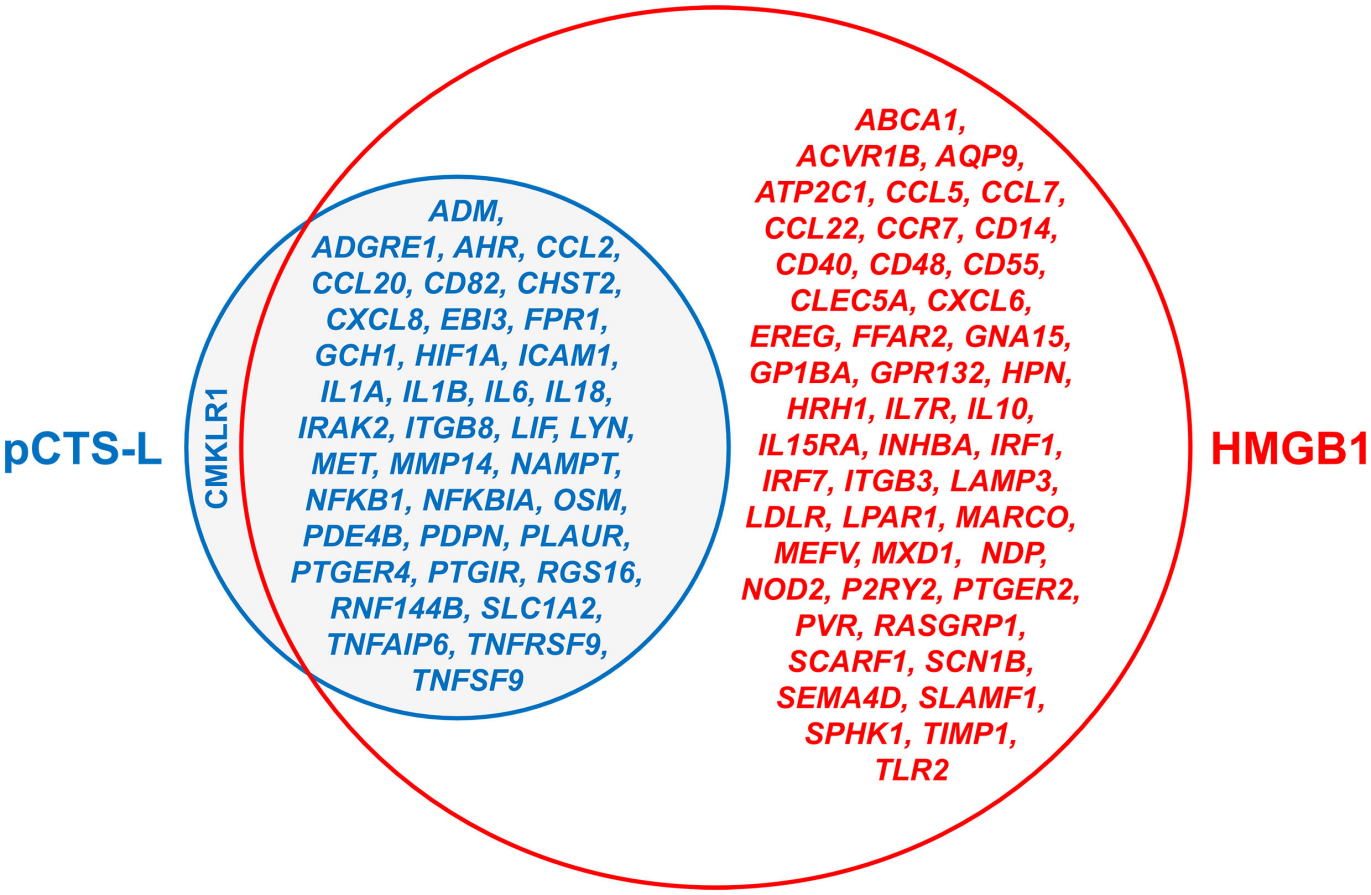
Venn diagram of inflammatory genes upregulated by HMGB1 and pCTS-L. A Venn diagram illustrating the unique and overlapping sets of genes significantly upregulated following stimulation with HMGB1 (0.5 µg/ml) and pCTS-L (2.0 µg/ml) in human PBMCs. The large red circle encompasses all 85 genes upregulated by HMGB1. The smaller blue circle represents the 39 genes upregulated by pCTS-L, including all 38 genes in the central overlapping region that are commonly upregulated by both HMGB1 and pCTS-L. The genes listed in red font, outside the overlap but within the red circle, represent 47 genes uniquely upregulated by HMGB1.

RNA-seq analyses confirmed that both HMGB1 and pCTS-L elicit robust inflammatory responses in human PBMCs, upregulating core pro-inflammatory cytokines (e.g., *IL1A, IL1B, IL4I1*, and *IL6*), chemokines (e.g., *CCL2, CCL3, CCL4, CCL4L2, CCL7, CCL8/MCP1, CCL18, CCL20, CXCL1/GRO-α*, and *CXCL8/IL-8*), S100 proteins (e.g., S100A8, S100A9, S100A12), interferons (e.g., *IFNA, IFNB*, and *IFNG*), and metallothionein genes (e.g., *MT1* and *MT2A*) (**Figure 3**) (101). These findings mirrored our previous observations that both HMGB1 and pCTS-L induce various cytokines (e.g., TNF and IL-6) and chemokines (e.g., CXCL5/ENA78, CXCL1/GRO-α, CXCL8/IL-8, CCL2/MCP-1, CCL5/RANTES, and CCL7/MCP-3) in human PBMCs (6, 102). Mechanistically, both mediators converge on activating the non-canonical NF-κB pathway, evidenced by NFKB2 and RELB upregulation (101). Unlike the canonical NF-κB pathway, which is rapid and primarily involved in immediate inflammatory responses, the non-canonical NF-κB pathway is generally slower, more sustained (103), and contributes to the pathogenesis of various inflammatory and autoimmune diseases, including sepsis and RA (104–106). Our findings are consistent with previous reports that HMGB1 stimulates leukocyte migration through the IKKα-dependent NF-κB p52/RelB non-canonical pathway (107).

Despite these commonalities, HMGB1 demonstrates a superior capacity to orchestrate inflammation. At a lower concentration (0.5 µg/ml), HMGB1 triggered nearly four times more differentially expressed genes than pCTS-L (2.0 µg/ml), signifying a broader transcriptional alteration (101). This DEG count, however, solely reflects the breadth of transcriptional alteration, and it does not fully equate to the extent of biological responses. Crucially, HMGB1 uniquely upregulates *CASP4* and *CASP5* (functional homologs of mouse *CASP11*) (101), components essential for the non-canonical inflammasome pathway, which leads to pyroptotic cell death and release of inflammasome-dependent cytokines (e.g., IL-1β, IL-18, and HMGB1) (33, 108, 109). This finding aligns with the observations that HMGB1 triggers CASP-11-dependent inflammasome activation and macrophage pyroptosis (17, 54). In murine peritoneal macrophages, pCTS-L also induced pro-Casp-11 expression and CASP-11 maturation, supporting a similar role for pCTS-L in CASP-11-associated pyroptosis (6).

Furthermore, HMGB1 induces a wider array of cytokines (e.g., *IL23A, IL24, IL10, IL36G, IL7, IL7R, HGF, IL6ST, IL11RA, IL1RL2, IL16, IL2RA, IFITM10, IL15RA*, and *IL18BP*) and chemokines (e.g., *CXCL5, CXCL13, CXCL16, CXCR2, CCL22*, and *CCL23*) (**Figure 3**) (101). Notably, cathepsin L (*Ctsl*) itself was also upregulated by HMGB1 (100, 101), suggesting potential feedforward loops or cross-talk between these two inflammatory mediators. This findings established a novel HMGB1-pCTS-L axis (100, 101) by showing that HMGB1—a key driver of both sepsis (5, 16, 17, 110) and RA (18–22)—triggers pCTS-L expression and release in human immune cells. Our findings delineate the distinct yet overlapping roles of HMGB1 and pCTS-L in orchestrating inflammatory responses (**Figure 3**), offering a foundation for targeted therapeutic development for inflammatory diseases.

## 5 Discovery of tetranectin as a dual regulator of HMGB1-mediated responses

The host possesses an intricate network of endogenous counter-regulatory proteins that finely tune the extracellular release and pro-inflammatory actions of HMGB1. For instance, thrombomodulin (TM) can bind HMGB1 to interrupt its engagement with receptors (111), thereby limiting inflammatory signaling (112, 113). Consistently, it reduces animal mortality in animal models of sepsis (114, 115), endotoxemia (112), and colitis (116). Similarly, Haptoglobin (HP) sequesters HMGB1 and facilitates the CD163-dependent endocytosis of HMGB1/HP complexes, shifting immune responses towards an anti-inflammatory phenotype, characterized by the production of heme oxygenase-1 and IL-10 (**Figure 1B**, **Table 1**) (117). Complement component factor 1q (C1q) also interacts with HMGB1, similarly promoting the production of anti-inflammatory cytokines (e.g., IL-10, **Figure 1B**) (118–120).

### 5.1 TN as a positive regulator of HMGB1-mediated pyroptosis

Our investigation into endogenous proteins modulating HMGB1 release revealed some intricate roles for TN in sepsis. These findings originated from observing a near-complete depletion of a 20-kDa protein in the blood of a sepsis patient who succumbed to the disease (121). Subsequent identification through mass spectrometry and immunoblotting confirmed this protein as human TN. Clinical studies corroborated this observation, showing a 60–70% reduction in plasma TN levels in septic patients, a decline that resembled a significant decrease in experimental sepsis models (121). These findings also mirrored with other clinical observations that TN levels similarly decreased in inflammatory diseases such as COVID-19 (122), RA (123, 124), coronary artery disease (125), heart failure (126, 127), and renal diseases (128). Surprisingly, a hyperglycemia-induced elevation of TN expression was observed in adipocytes of patients and animals with diabetes (129), suggesting a paradoxically harmful role of TN under certain conditions.

**Figure 4:**
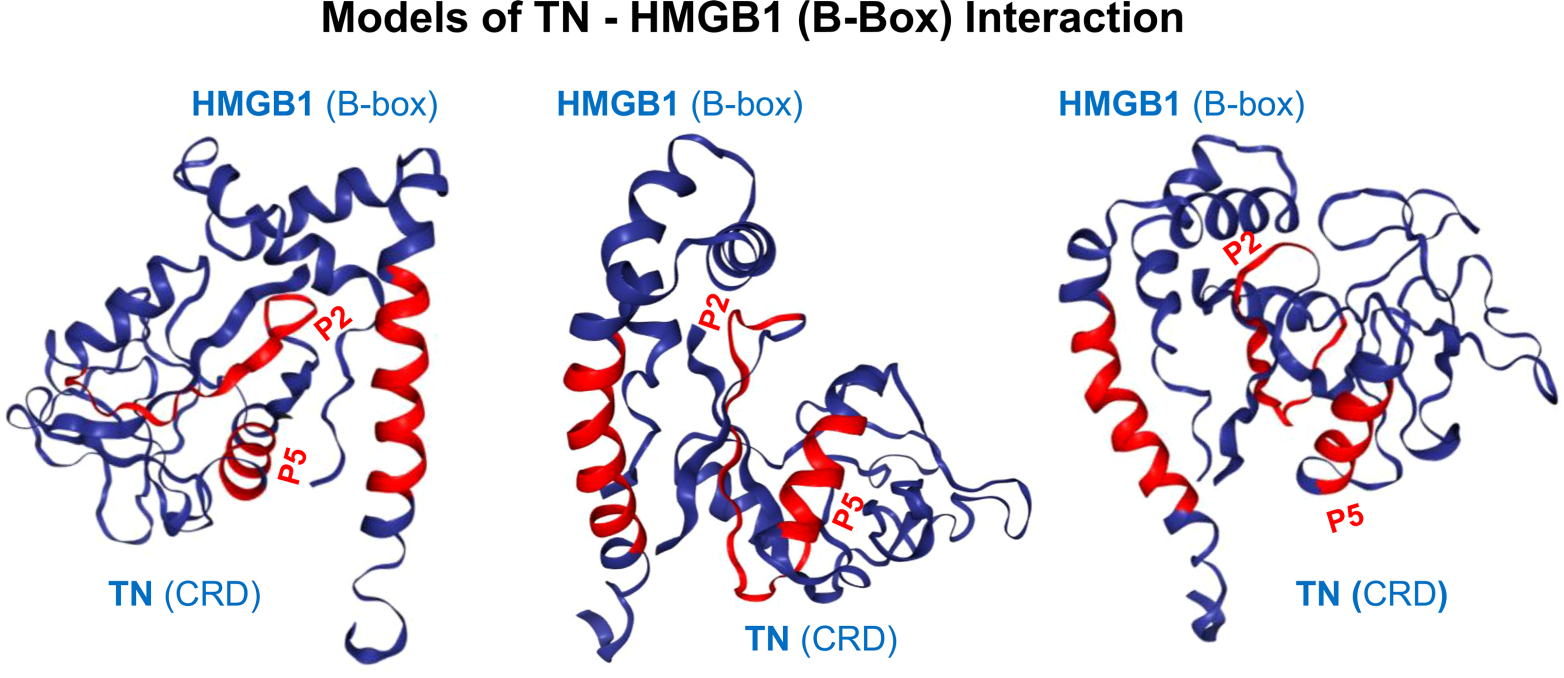
Proposed models for tetranectin-HMGB1 interaction. Molecular docking suggests that HMGB1 may bind TN through regions encompassing both the P2 and P5 peptides (highlighted in red to illustrate their relative positions), which are spatially adjacent on the surface of the TN protein. The binding of P2- or P5-reactive antibodies would sterically hinder HMGB1’s access to these regions, thereby physically obstructing the TN-HMGB1 interaction. While the P2 peptide is known to bind HMGB1 directly, it remains unknown whether the isolated P5 peptide can similarly bind HMGB1 as well.

**Figure 5:**
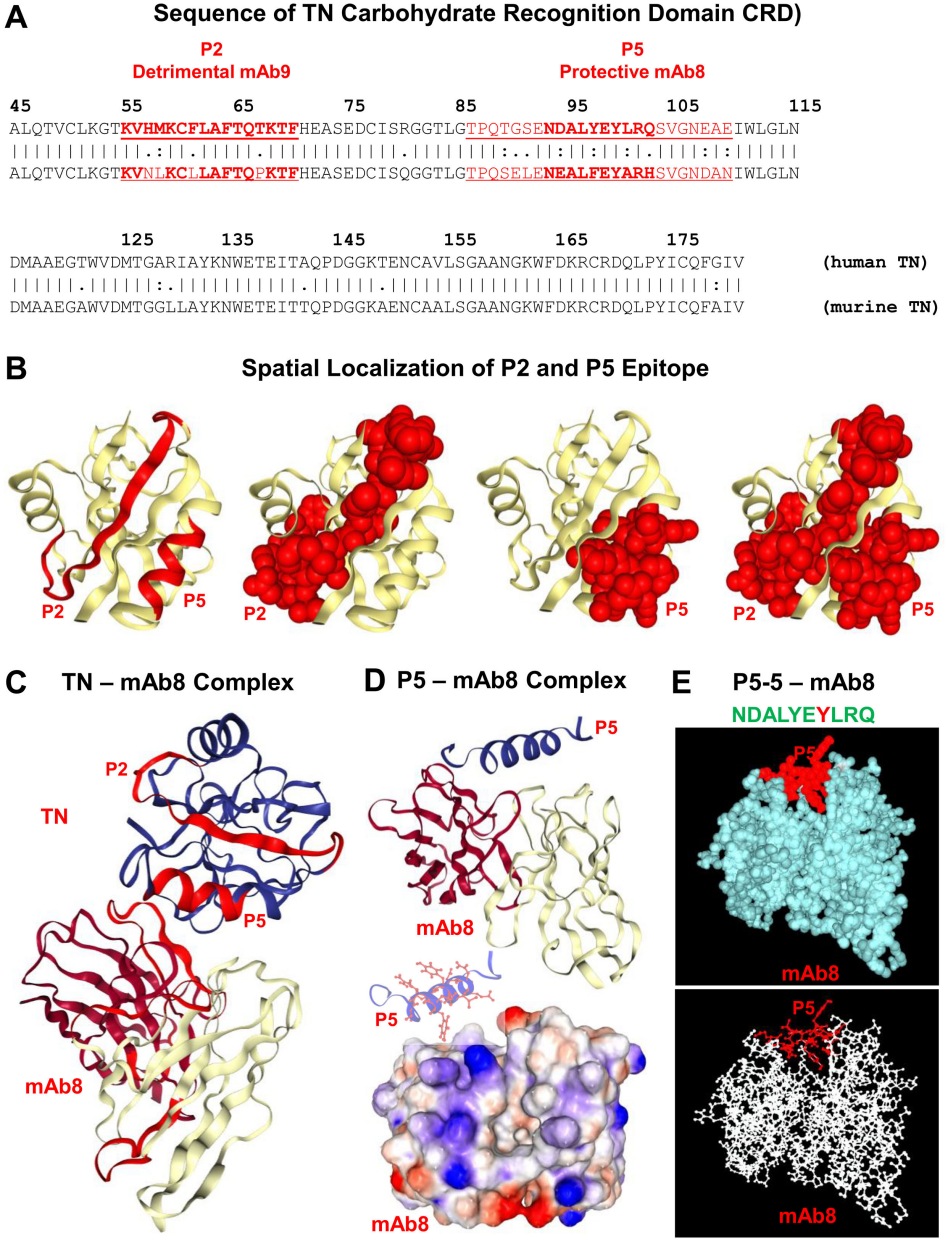
Structural models TN CRD-mAb8 interactions. **(A)** Amino acid sequence of human and murine TN C-terminal carbohydrate recognition domain (CRD), highlighting the P2 and P5 epitopes targeted by the detrimental mAb9 and protective mAb8, respectively. **(B)** Three-dimensional (3-D) structural models of the TN CRD, illustrating the spatial arrangement and surface accessibility of P2 and P5 peptide sequences within the protein. **(C)** Molecular docking of the TN with mAb8, highlighting how mAb8 binds to the P5 epitope within the TN protein’s CRD. The 3-D structure was predicted using Machine Learning-based antibody modelling (ABodyBuilder-ML), followed by the employment of the ClusPro Web Server to predict potential structures of TN CRD/mAb8 complexes. The full TN CRD is depicted in blue, with P2 and P5 shown in red to illustrate their relative positions, while the P5-reactive mAb8 is presented in light yellow and dark red. This interaction suggests that mAb8 may sterically hinder TN’s interactions with other proteins (e.g., HMGB1) through a region around the P5 epitope. **(D)** Molecular docking of mAb8 with the isolated P5 peptide. The ClusPro Web Server was used to predict possible structures of P5/mAb8 complexes. This demonstrates that the P5 peptide maintains a consistent orientation when bound to mAb8, whether as part of the full TN CRD or in isolation. Detailed representations of the P5 peptide (blue) docked onto mAb8 are provided through both ribbon and electrostatic surface models. **(E)** Molecular docking of mAb8 with the P5–5 epitope peptide (NDALYEYLRQ). Detailed representations of the P5–5 peptide (red) docked onto mAb8 are presented using both ribbon and ball-and-stick models, illustrating how the P5–5 epitope fits into mAb8’s P5-binding pocket.

This sepsis-associated TN depletion was attributed to its ability to capture extracellular HMGB1 (**Figure 4**), thereby facilitating the endocytosis of TN/HMGB1 complexes by innate immune cells (121). Consistent with prior observations that HMGB1 endocytosis triggers macrophage pyroptosis (17, 54), we discovered that the TN-mediated HMGB1 uptake promoted macrophage pyroptosis and immunosuppression that may compromise host immunity against infections (**Figure 1B**) (121). This novel “guilt-by-association” paradigm has opened new therapeutic avenues, leading to the development of TN P5 peptide (NDALYEYLRQ)-specific mAb8 (**Figures 5A–E**) capable of disrupting the detrimental HMGB1/TN interaction (121, 130–132). Pre-treatment of TN with mAb8 led to an approximately 85% reduction in HMGB1 binding and a six-fold increase in the equilibrium dissociation constant (K_D_) (121). Consequently, mAb8 prevented the reciprocal enhancement of HMGB1 and TN cellular uptake, and blocked TN/HMGB1-induced ASC translocation/aggregation and macrophage pyroptosis (121). Thus, mAb8 may sterically hinder the interaction between TN and HMGB1 by engaging its P5 epitope. This effect likely extends to adjacent epitopes, such as P2, particularly due to their spatial proximity within the three-dimensional structure (**Figures 5B, C**), despite their linear distance (**Figure 5A**).

In an animal model of sepsis, these TN P5-specific mAbs significantly attenuated sepsis-induced lethality (**Table 1**), thereby highlighting their therapeutic potential in preventing detrimental TN/HMGB1 interactions. Recently, another TN P5-reactive mAb12F1 has been developed (133), demonstrating similar protection in an animal model of lethal endotoxemia (134) (**Table 1**). Given the 100% sequence identity of the P5 peptide (NDALYEYLRQ) across humans and numerous other mammalian species (e.g., baboon, bovine, cougar, elephant, monkey, pig, and tiger) (121), these P5-reactive protective mAbs hold promising potential for the clinical management of inflammatory diseases.

### 5.2 TN as a negative regulator of HMGB1 release

Beyond its involvement in regulating HMGB1-induced pyroptosis, TN also serves as a negative regulator of HMGB1 release, thereby exhibiting a paradoxical dual functionality. Specifically, highly purified TN selectively inhibited LPS- or SAA-induced HMGB1 release without affecting the secretion of other cytokines and chemokines (121). This suppression was attributed to TN’s ability to capture extracellular HMGB1 (**Figure 4**) and facilitate the endocytosis of TN/HMGB1 complexes by macrophages (**Figure 1B**), thereby clearing HMGB1 from the circulation to exert a protective role. Indeed, genetic disruption of TN expression increased susceptibility to lethal sepsis, leading to exacerbated inflammation and injury (121). Conversely, supplementing septic animals with recombinant TN protein at physiological concentrations conferred dose-dependent protection (**Table 1**).

Structurally, TN features an N-terminal heparin binding and α-helix trimerization segment, and a C-terminal carbohydrate recognition domain (CRD, **Figure 5A**) that enables binding to plasminogen (135, 136), apolipoprotein A1 (137), tissue-type plasminogen activator (t-PA) (138), and HMGB1 (121). To identify the essential protective region of TN, we generated a mutant (ΔTN) lacking the N-terminal heparin-binding and α-helix trimerization regions (100). Comprising the C-terminal CRD (residues 45-181) (**Figure 5A**), this ΔTN mutant conferred protection against lethal sepsis (100) comparable to that of the full-length TN (residues 1-181, **Table 1**) (121), thereby localizing the protective region within its C-terminal CRD.

### 5.3 Balanced actions of TN in diverse pathological conditions

Under normal physiological conditions, circulating TN levels are relatively high, while extracellular HMGB1 levels are very low or negligible, ensuring that TN does not exert significant effects on extracellular HMGB1 function. Upon inflammatory insults, circulating HMGB1 levels begin to rise due to cellular secretion and/or release. Meanwhile, circulating TN levels decrease during acute (121, 122) or chronic (123, 124) inflammation, thereby gradually reducing the TN: HMGB1 ratio as inflammation progresses. The reason for this decrease in TN could be multifaceted, possibly involving proteolytic degradation, consumption through binding to various ligands (including HMGB1) (121), or tissue redistribution.

This dynamic shift in the TN: HMGB1 ratio might be central to its dual functionality. Initially, at a higher TN: HMGB1 ratio, TN may predominantly act as a sequestering agent by directly binding to extracellular HMGB1 to form TN-HMGB1 complexes. This binding likely prevents free HMGB1 from interacting with its cognate pro-inflammatory receptors (e.g., TLR2, TLR4, or RAGE) on immune cells. By sequestering and potentially facilitating the clearance of extracellular HMGB1, TN helps to dampen the initial inflammatory response, thereby exerting a net negative regulatory effect on HMGB1-mediated inflammation. As inflammation progresses and the TN: HMGB1 ratio decreases, TN’s role may shift to a “booster” for HMGB1’s activity, particularly promoting specific cellular uptake pathways that lead to pyroptosis. Mechanistically, this could involve the facilitated endocytosis of the TN-HMGB1 complex through receptors for both TN and HMGB1 on target cells, which synergistically promote the efficient internalization of the TN-HMGB1 complexes.

In the early phases of acute sepsis, massive and rapid HMGB1 release can be detrimental. If TN is highly expressed and can effectively sequester and neutralize the surge of HMGB1, it would act as a protective, negative regulator, helping to dampen the hyperinflammatory response. However, if TN levels are rapidly depleted, the subsequent shift to a low TN: HMGB1 ratio, coupled with TN’s positive regulatory mechanisms (e.g., enhanced endocytosis leading to pyroptosis), could exacerbate the pathology by facilitating HMGB1-mediated pyroptosis and immunosuppression. In this scenario, the initial negative regulation is lost, allowing HMGB1’s detrimental effects to flourish, potentially enhanced by TN’s altered role. Additionally, the expression pattern of TN varies across tissues (121), with the lung being a major source under normal healthy conditions. This spatial distribution suggests that TN may serve as a crucial local regulator of HMGB1 function in certain organs such as the lung, particularly in the context of infections, acute lung injury, or other localized inflammatory processes. For instance, in the lung, a high local concentration of TN could initially protect against HMGB1-mediated damage, while a decline could lead to enhanced HMGB1 signaling. Thus, the fascinating dual roles of TN in modulating HMGB1 responses underscore the complexity of endogenous regulatory mechanisms. We hypothesize that the balance between TN’s positive and negative regulatory actions may be determined by a sophisticated interplay of TN: HMGB1 concentration dynamics and the cellular and pathological microenvironment. Future research needs to employ advanced methodologies, such as cellular co-culture models and *in vivo* studies in various disease models to fully elucidate the conditions under which one role predominates over the other. This mechanistic understanding is crucial for harnessing the therapeutic potential of targeting the TN-HMGB1 axis.

### 5.4 Development of TN-derived peptide as a specific inhibitor of the HMGB1-pCTS-L axis

In agreement with TN’s protective role, we discovered some TN-specific mAbs worsened the outcome of lethal sepsis (121). Using a panel of ten peptides (P1–P10) spanning TN’s CRD region, we mapped the epitope of these detrimental mAbs (e.g., mAb9) to the P2 peptide (**Figure 5A**). We hypothesized that the P2 epitope itself, when delivered as a peptide agonist, could function as a molecular interceptor that can directly bind HMGB1 to interfere with its pathogenic actions (**Figure 1B**). Indeed, surface plasmon resonance (SPR) confirmed P2-1’s high-affinity binding to HMGB1 (K_D_ = 16.87 nM) (100), primarily within the pro-inflammatory B-box of HMGB1 (**Figure 1A**) (139). These P2-binding sites (E131, N134, K152, and E156) were spatially distant from the TLR4-binding domain (residues 89–108), but partially overlapped with the RAGE-binding region (residues 150–183, **Figure 1A**) (100, 140). This unique spatial geometry suggests that P2–1 does not interfere with HMGB1-TLR4 interactions (100), thereby preserving TLR4-dependent cytokine and chemokine responses (**Figure 1B**) (44, 139). Consistently, RNA-seq analysis confirmed that P2–1 did not broadly diminish HMGB1-induced cytokine and chemokine production (100). However, it specifically inhibited HMGB1-induced *Ctsl* mRNA expression and pCTS-L release, thereby establishing itself as a highly specific modulator of the HMGB1-pCTS-L axis (**Figure 1B**) (100).

Specifically, P2–1 potentially disrupts HMGB1-RAGE signaling (100), a pathway critical for HMGB1 internalization and macrophage pyroptosis (**Figure 1B**) (17, 54). In contrast to TN (121), P2–1 paradoxically reduced HMGB1 uptake and significantly inhibited HMGB1-induced macrophage pyroptosis (**Figure 1B**), as evidenced by reduced trypan blue uptake and LDH release (100). Thus, a striking contrast exists between full-length TN and P2–1 regarding their modulation of HMGB1-driven macrophage dysfunctions. The full-length TN, when complexed with HMGB1, may interact with as-yet-unidentified macrophage surface receptors, thereby stimulating receptor-mediated internalization of TN/HMGB1 complexes to trigger pro-pyroptotic pathways (**Figure 1B**). In contrast, P2–1 maintains its HMGB1-binding capacity but lacks the necessary structural domains to engage these endocytosis-promoting macrophage receptors (**Figure 1B**, **Table 1**).

This divergent mechanism positions P2–1 as a superior candidate for specifically countering HMGB1-mediated deleterious processes, such as pCTS-L upregulation and macrophage pyroptosis (**Figure 1B**) (100). Specifically, P2–1 curtails HMGB1-driven *Ctsl* mRNA expression and subsequent pCTS-L release, establishing it as a highly specific inhibitor of the HMGB1-pCTS-L axis (**Figure 1B**) (100). It is currently unclear if this selective RAGE antagonism fully explains how P2–1 precisely inhibits HMGB1-induced pCTS-L synthesis (**Figure 1B**), and will thus be important to investigate whether P2-1’s selective pCTS-L inhibition depends on blocking RAGE or other, as-yet-unidentified receptors.

Notably, bioinformatics analyses (e.g., BLAST searches) revealed that the HMGB1 target sequence (i.e., KKLGEMWNNTAADDKQPYEKKAAKLKEKYEKDIAAYR) for the P2–1 peptide shares significant sequence homology with both HMGB1 and HMGB2 proteins, indicating a potential for off-target interactions with HMGB2. However, given that extracellular HMGB2 is less potently inflammatory than HMGB1 (141), any potential off-target interaction of P2–1 with HMGB2 may not yield substantial biological consequences. Nevertheless, future studies are necessary to comprehensively screen for unintended protein binding partners or pathway modulations in complex biological matrices or *in vivo*.

### 5.5 Therapeutic potential of TN-derived P2–1 peptide in sepsis and RA

In an animal model of sepsis, repeated administration of P2 significantly increased survival (100). To improve solubility and oxidative resistance, we engineered a derivative, P2-1, through specific amino acid substitutions (**Figure 1A**). Notably, P2–1 demonstrated potent protection even with delayed administration (24 h post-CLP) and at a dose five-fold lower than P2 (**Table 1**) (100). Meanwhile, it significantly reduced sepsis-induced systemic accumulation of a wide array of proinflammatory cytokines (e.g., IL-6) and chemokines (e.g., CXCL13/BLC, G-CSF, CXCL1/KC/GRO-α, MCP-1, MIP-1γ, CXCL3/MIP-2/GRO-β, and sTNFRI) (100), a profile that closely resembled the effect of pCTS-L-neutralizing antibodies in sepsis (6). It suggests that P2–1 confers protection against sepsis potentially by mitigating pCTS-L-mediated production of key surrogate biomarkers (142, 143).

In an animal model of CAIA-induced RA, P2–1 similarly reduced disease severity, pain sensitivity, and inflammatory markers (sTNFRI, sTNFRII, MIP-1γ/CCL9/10, and VCAM-1) within the inflamed joint tissue (100). Given pCTS-L’s elevation in experimental (92–94) and clinical arthritis (95–97), we also tested a pCTS-L-neutralizing mAb2 in this CAIA model. Consistently, mAb2 significantly mitigated arthritis severity and pain sensitivity (**Table 1**) (100), validating pCTS-L as a therapeutic target. Furthermore, these findings paralleled P2-1’s efficacy in specifically inhibiting the HMGB1-pCTS-L inflammatory axis *in vitro*, and conferring protection against both sepsis and RA *in vivo* (100).

## 6 Conclusions and future perspectives

Although HMGB1 is generally not inducible, it can be actively secreted through non-classical ER-Golgi pathways or passively released from cells undergoing necrosis or pyroptosis. Upon release, extracellular HMGB1 binds to TLR4 to promote cytokine/chemokine production, and to RAGE to induce pyroptosis and immunosuppression, thereby serving as a critical mediator of lethal sepsis and RA. In contrast, pCTS-L is induced by inflammatory stimuli (e.g., LPS, IFN-γ, SAA, and HMGB1) and can be actively secreted via the classical ER-Golgi pathway. Similarly, extracellular pCTS-L binds to both TLR4 and RAGE to induce cytokines/chemokines, hepatic fibrinogen-α/β/γ, and CASP-11, thereby serving as another critical mediator of lethal sepsis and RA. Moreover, both HMGB1 and pCTS-L induce NFKB2 and RELB expression, thereby activating the non-canonical NF-κB pathway, leading to generally slower and more sustained signaling in sepsis and RA. Notably, HMGB1 can upregulate pCTS-L, thereby establishing a pathogenic HMGB1-pCTS-L axis in sepsis and RA. The relatively delayed onset of the HMGB1-pCTS-L axis during the course of many inflammatory diseases makes it a more practical therapeutic target in clinical settings, particularly in comparison with some earlier inflammatory cascades.

TN simultaneously acts as both a negative regulator of HMGB1 release and a positive regulator of HMGB1-induced pyroptosis. This intricate balance underscores TN’s pivotal, albeit double-edged, role in inflammatory diseases, offering new targets for therapeutic interventions. On one hand, we have generated a panel of TN P5-specific mAbs to disrupt the harmful TN-HMGB1 interaction, thereby conferring protection against lethal sepsis. On the other hand, we have also developed mimetic peptide-based therapeutics for both sepsis and RA by leveraging the epitope of certain TN P2-specific detrimental mAbs as specific inhibitor of the HMGB1-pCTS-L axis. This specific suppression of the pathogenic HMGB1-pCTS-L axis provides a sophisticated immunomodulatory strategy that avoids compromising beneficial host inflammatory responses. These therapeutic agents achieve their therapeutic selectivity by suppressing HMGB1-mediated harmful actions exclusively at pathological sites, since HMGB1 is typically confined to the nucleus under normal physiological conditions, making it inaccessible to HMGB1-targeting therapeutics. This inherent “disease-triggered” activation mechanism ensures that their immunomodulatory effects are confined to inflamed tissues, potentially offering a more favorable safety profile compared to broad-acting systemic immunosuppressive agents.

For RA, P2–1 addresses significant drawbacks of current anti-TNF biologics, specifically systemic immune suppression and lack of patient response. By focusing on an unexplored downstream pathway, it presents a novel treatment avenue for patients resistant to other therapies. Thus, P2–1 concurrently stands out as a highly promising next-generation therapy for RA and a more precise intervention for sepsis, highlighting its wide-ranging clinical applicability. Beyond its role in sepsis and RA, HMGB1 has also been implicated in the pathogenesis of numerous other autoimmune diseases (144), including inflammatory bowel disease (IBD) (145, 146), multiple sclerosis (147), systemic lupus erythematosus (SLE) (148–151), and vitiligo (152–154). Therefore, it will be important to explore the therapeutic efficacy of P2–1 in the treatment of various autoimmune diseases.

Although clinical trials involving TLR4 ligand antagonists (e.g., LPS mimetics, eritoran) (155) and early cytokines (e.g., TNF) neutralizing mAbs (156, 157) failed to improve sepsis survival, it is crucial to distinguish between targeting general TLR4 activation or early cytokines, and specifically interfering with HMGB1/pCTS-L-TLR4 interactions. Several factors might explain the disparities observed in therapeutic outcomes, including kinetic differences of ligands, optimal timing of intervention, and targeting specificity. For instance, LPS, a PAMP rapidly released from Gram-negative bacteria, along with early cytokines (e.g., TNF), drive the initial, acute phase of the inflammatory response in sepsis (**Figure 2**). In contrast, HMGB1 and pCTS-L are released later in the course of sepsis, typically hours to days after the initial insult (**Figure 2**), thereby contributing to persistent inflammation and immunosuppression. Previous clinical trials that targeted TLR4 or early cytokines, often using LPS mimetics or cytokine-neutralizing antibodies, intervened very early in the course of sepsis. Blocking all TLR4 signaling and early cytokines at this acute stage may have been detrimental, as TLR4 and early cytokines are crucial for initiating protective immune responses essential for pathogen clearance. Given the delayed release of HMGB1 and pCTS-L, targeting HMGB1/pCTS-L-TLR4 interaction could offer a wider therapeutic window (**Figure 2**). This would potentially allow for intervention in patients progressing to a more persistent inflammatory state or organ dysfunction, without compromising crucial early host defense.

Furthermore, TLR4 recognizes a diverse array of ligands beyond LPS, HMGB1, and pCTS-L. Consequently, a broad blockade of TLR4 could inadvertently interfere with beneficial or compensatory signaling pathways. In contrast, specifically targeting the HMGB1/pCTS-L-TLR4 axis, potentially through HMGB1/pCTS-L-neutralizing mAbs, provides a more precise disruption of a delayed inflammatory pathway. This presents a more refined therapeutic strategy compared to generalized TLR4 antagonism. Alternatively, distinct approaches, particularly those targeting the HMGB1-RAGE-pCTS-L axis (with P2-1, for instance), could facilitate more precise intervention in delayed signaling pathways for patients progressing into a persistent immunosuppression state, without compromising crucial early host defense mechanisms.

However, translating these neutralizing mAbs into clinical therapies needs to overcome two principal hurdles: immunogenicity and pharmacokinetics. For these neutralizing mAbs, humanization is essential to reduce their immunogenicity before human use. Similarly, targeting the HMGB1-pCTS-L axis with peptide-based therapeutics also faces several translational challenges, including rapid proteolytic degradation (by peptidases and proteases) and chemical instability (e.g., oxidation, deamidation, and aggregation). To address these issues and enhance stability, various strategies may be employed. These include amino acid modifications (such as incorporating D-amino acids or non-natural amino acids) to resist enzymatic cleavage, cyclization (forming cyclic peptides) to increase rigidity, and N- and C-terminal modifications (e.g., acetylation or amidation) to protect against exopeptidase activity. Additionally, other chemical modifications (e.g., PEGylation) might be required to increase hydrodynamic size, reduce renal clearance, and minimize proteolytic degradation. Furthermore, direct injection or topical applications, where applicable, can be adopted to maximize local concentration while minimizing systemic exposure.

Despite their generally lower immunogenicity compared to larger protein biologics, peptide therapeutics can still elicit an immune response upon repeated administration. This can lead to the production of neutralizing antibodies, which may alter the pharmacokinetics of therapeutic peptides, thereby reducing therapeutic efficacy and potentially leading to adverse consequences, much like those observed with the TN P2-reactive mAb9. To mitigate immunogenicity, strategies such as sequence optimization through “de-immunization” design (to remove predicted T-cell epitopes), selection of less immunogenic scaffolds, and the use of immunomodulatory excipients or conjugation techniques like PEGylation (which can shield immunogenic regions) may be employed. Therefore, it is crucial to conduct exhaustive preclinical immunogenicity testing during development to predict and manage potential immune responses effectively. Following these preclinical optimizations, clinical development will proceed from Phase 1 safety assessments in healthy individuals to Phase 2 efficacy trials in sepsis or RA patients displaying elevated HMGB1 and pCTS-L biomarkers.

## Glossary

C1q: complement component factor 1q
CAIA: Collagen antibody-induced arthritis
CLP: Cecal ligation and puncture
CRD: Carbohydrate recognition domain
Ctsl: Cathepsin L
DAMP: damage-associated molecular pattern
ER: endoplasmic reticulum
HMGB1: High mobility group box 1
HP: haptoglobin
K_D_: equilibrium dissociation constant
LPS: Lipopolysaccharides
mAb: Monoclonal antibody
MCP-1: Monocyte chemoattractant protein-1
PAMP: pathogen-associated molecular patterns
PBMCs: Human peripheral blood mononuclear cells
pCTS-L: procathepsin L
RA: Rheumatoid arthritis
RAGE: Receptor for advanced glycation end products
SARS-CoV-2: severe acute respiratory syndrome (SARS)-like coronavirus 2
SPR: Surface plasmon resonance
TLR4: toll-like receptor 4
TN: Tetranectin
